# Euglycemic ketoacidosis in a nondiabetic patient receiving dapagliflozin for heart failure

**DOI:** 10.1210/jcemcr/luag275

**Published:** 2026-09-11

**Authors:** Giullia Silveira, Yuliana Kvasha, Preksha Shah, Harin Shah, Jyothi Gogineni

**Affiliations:** Department of Internal Medicine, Northwestern Medicine McHenry Hospital, McHenry, IL 60050, USA; Department of Internal Medicine, Northwestern Medicine McHenry Hospital, McHenry, IL 60050, USA; Department of Internal Medicine, Northwestern Medicine McHenry Hospital, McHenry, IL 60050, USA; Department of Internal Medicine, Northwestern Medicine McHenry Hospital, McHenry, IL 60050, USA; Department of Endocrinology, Northwestern Medicine McHenry Hospital, McHenry, IL 60050, USA

**Keywords:** euglycemic ketoacidosis, ketoacidosis, SGLT-2, dapagliflozin, heart failure

## Abstract

Sodium–glucose cotransporter 2 (SGLT2) inhibitors are increasingly used for cardiovascular indications in patients without diabetes, increasing exposure to uncommon metabolic complications. Euglycemic ketoacidosis is a serious but under-recognized adverse effect that may be overlooked because marked hyperglycemia is absent. We report a 72-year-old woman without diabetes who presented with high anion gap metabolic acidosis: bicarbonate 16 mmol/L (reference range, 22-29 mmol/L), anion gap 19 mmol/L (reference range, 8-12 mmol/L), and glucose 140 mg/dL (7.8 mmol/L) (reference range, 70-100 mg/dL [3.9-5.6 mmol/L]). Serum beta-hydroxybutyrate was 3.4 mmol/L (reference range, <0.5 mmol/L), confirming ketosis. She was receiving dapagliflozin for heart failure. Discontinuation of dapagliflozin, intravenous fluids, and oral carbohydrate supplementation resulted in rapid resolution without insulin therapy. Because no alternative precipitant was identified and recurrence remained a concern, dapagliflozin was permanently discontinued, and SGLT2 inhibitor rechallenge was not recommended. This case demonstrates that euglycemic ketoacidosis may occur in nondiabetic patients without typical precipitants. Early recognition and individualized management can prevent unnecessary escalation of care as SGLT2 inhibitor use expands.

## Introduction

Sodium–glucose cotransporter 2 (SGLT2) inhibitors have demonstrated significant cardiovascular and renal benefits in patients with heart failure and chronic kidney disease [1, 2]. As their use expands beyond diabetes, recognition of uncommon adverse effects such as euglycemic ketoacidosis becomes increasingly important [3].

## Case presentation

A 72-year-old woman with a history of ischemic cardiomyopathy with reduced ejection fraction (30%), coronary artery disease status post percutaneous coronary intervention, and prior transcatheter aortic valve replacement presented to the emergency department with several days of persistent tachycardia and chest discomfort. She described a sensation of “unpleasant chest awareness” associated with sustained elevated heart rates recorded on her wearable device. She denied fever, recent illness, reduced oral intake, nausea, vomiting, abdominal pain, alcohol use, ketogenic diet, recent dietary change, recent surgery, recent travel, vaccination, or immunotherapy. There had been no recent initiation of a glucagon-like peptide-1 receptor agonist or other new medication associated with reduced intake or ketosis.

Her home medications included guideline-directed medical therapy for heart failure, including dapagliflozin, which had been initiated ∼6 months prior. She had no prior history of diabetes mellitus or impaired glucose tolerance and had not experienced similar symptoms previously. Hemoglobin A1c before dapagliflozin initiation was 5.4% (36 mmol/mol) (reference range, <5.7% [<39 mmol/mol]), with preserved kidney function before initiation, with creatinine 0.74 mg/dL (65.4 µmol/L) (reference range, 0.6-1.1 mg/dL [53-97 µmol/L]) and estimated glomerular filtration rate 86 mL/min/1.73 m^2^ (reference range, ≥60 mL/min/1.73 m^2^). She had no known thyroid disease, autoimmune disease, pancreatic pathology, or family history of diabetes or autoimmune disease.

Her body mass index at presentation was 20.9 kg/m^2^, with a weight of 128 lb (58.1 kg). Approximately 6 months earlier, her weight was 133 lb (60.3 kg), corresponding to a body mass index of 21.5 kg/m2; therefore, there was no remarkable interval change in body mass index.

On presentation, she was tachycardic with heart rates ranging from 150 to 170 beats per minute. Blood pressure was stable, and oxygen saturation was normal on room air. Physical examination was notable for tachycardia without signs of volume depletion, infection, or altered mental status. Initial evaluation focused on cardiac etiologies, including arrhythmia and demand ischemia, given her significant cardiovascular history.

## Diagnostic assessment

Laboratory evaluation demonstrated a bicarbonate level of 16 mEq/L (16 mmol/L) (reference range, 22-29 mEq/L [22-29 mmol/L]) and an elevated anion gap of 19 mEq/L (19 mmol/L) (reference range, 8-12 mEq/L [8-12 mmol/L]), consistent with high anion gap metabolic acidosis. Blood glucose was 140 mg/dL (7.8 mmol/L) (reference range, 70-100 mg/dL [3.9-5.6 mmol/L]), which was not consistent with typical diabetic ketoacidosis.

Given the discordance between metabolic acidosis and relatively normal glucose levels, further evaluation was pursued. Serum beta-hydroxybutyrate was elevated at 3.4 mmol/L (reference range, <0.5 mmol/L), and urinalysis confirmed ketonuria. Hemoglobin A1c was 5.5% (37 mmol/mol) (reference range, <5.7% [<39 mmol/mol]), supporting the absence of chronic hyperglycemia. There were no known factors to suggest an unreliable hemoglobin A1c, such as advanced kidney disease, recent transfusion, known hemoglobinopathy, or anemia. Available historical glucose measurements before admission ranged from 89 to 133 mg/dL (4.9-7.4 mmol/L). Insulin levels had not been obtained previously; therefore, homeostatic model assessment of insulin resistance could not be calculated.

To further exclude underlying diabetes or insulin deficiency, C-peptide was measured and found to be 3.0 ng/mL (1.0 nmol/L) (reference range, 0.8-3.1 ng/mL [0.26-1.03 nmol/L]), indicating preserved endogenous insulin secretion. Autoimmune markers including glutamic acid decarboxylase, islet cell, and zinc transporter 8 antibodies were negative.

Renal function was preserved on admission, as previously mentioned. The patient was hemodynamically stable, had no vomiting or poor intake, and had no clinical or laboratory evidence of dehydration.

Thyroid testing was obtained given the presenting tachycardia. Thyroid-stimulating hormone was 1.28 µIU/mL (1.28 mIU/L) (reference range, 0.30-5.33 µIU/mL [0.30-5.33 mIU/L]), and free thyroxine was 0.96 ng/dL (12.4 pmol/L) (reference range, 0.54-1.24 ng/dL [6.9-16.0 pmol/L]). A prior thyroid-stimulating hormone level was 1.49 µIU/mL (1.49 mIU/L), and she had no history of thyroid disease.

Liver testing did not suggest chronic liver disease. Her aspartate aminotransferase, alanine aminotransferase, alkaline phosphatase, and total bilirubin were all within normal limits. A Fibrosis-4 score calculated from available data was 2.38, an indeterminate value that should be interpreted cautiously in an older adult and in the absence of known chronic liver disease.

In the absence of other identifiable causes of ketosis, the clinical picture was most consistent with SGLT2 inhibitor-associated euglycemic ketoacidosis.

## Treatment

Dapagliflozin was discontinued on admission given concern for SGLT2 inhibitor-associated euglycemic ketoacidosis. The patient was initially managed with intravenous isotonic fluid resuscitation using normal saline to address possible subclinical volume depletion and facilitate renal clearance of ketones.

Given the absence of significant hyperglycemia and preserved endogenous insulin secretion, insulin therapy was deferred. Instead, oral carbohydrate supplementation was initiated, including administration of juice and scheduled bedtime snacks, with the goal of stimulating endogenous insulin release and suppressing ketogenesis.

Electrolytes were monitored closely, with particular attention to serum potassium, bicarbonate, and anion gap. No significant electrolyte abnormalities requiring correction were observed. Serial laboratory testing was performed to assess response to therapy.

## Outcome and follow-up

The patient demonstrated rapid biochemical improvement following initiation of supportive therapy. Within 24 hours, the anion gap decreased from 19 to 11 mmol/L (reference range, 8-12 mmol/L), and serum bicarbonate increased from 16 to 19 mmol/L (reference range, 22-29 mmol/L), consistent with resolution of metabolic acidosis. Serum beta-hydroxybutyrate levels declined in parallel, indicating suppression of ketogenesis, while blood glucose levels remained within the normal range.

Clinically, the patient remained stable without development of altered mental status, hemodynamic instability, or need for intensive monitoring. She did not experience complications related to treatment, including hypoglycemia or electrolyte disturbances.

Dapagliflozin was permanently discontinued at discharge. The patient was counseled regarding the risk of recurrent ketoacidosis with future SGLT2 inhibitor use and advised to avoid reinitiation of this medication class. Because the event occurred without diabetes, insulin deficiency, renal dysfunction, dehydration, diet change, illness, surgery, alcohol use, or another clear precipitant, decision was not rechallenge this patient with dapagliflozin or another SGLT2 inhibitor. Ongoing heart failure management was deferred to cardiology with emphasis on optimization of non-SGLT2 inhibitor guideline-directed medical therapy.

At short-term outpatient follow-up at 2 weeks, the patient remained asymptomatic, with no recurrence of metabolic abnormalities.

## Discussion

SGLT2 inhibitors have become a cornerstone of therapy for heart failure and chronic kidney disease, with demonstrated reductions in cardiovascular mortality and progression of renal disease [1, 2]. As their use expands beyond patients with diabetes, recognition of uncommon but clinically significant metabolic complications such as euglycemic ketoacidosis is increasingly important.

The pathophysiology of SGLT2 inhibitor-associated ketoacidosis is multifactorial. By promoting glycosuria, these agents reduce circulating glucose and insulin levels while increasing glucagon secretion, thereby shifting metabolism toward lipolysis and hepatic ketogenesis [3]. In parallel, urinary glucose loss prevents the degree of hyperglycemia typically seen in diabetic ketoacidosis, resulting in a “euglycemic” presentation that may delay diagnosis.

Observational studies have demonstrated an increased risk of ketoacidosis following initiation of SGLT2 inhibitors, even outside of traditional high-risk populations [4]. Postmarketing analyses have further confirmed this association, highlighting the importance of clinical vigilance [5]. More recent large-scale cohort studies provide additional insight into safety profiles in real-world populations, emphasizing that rare but serious adverse metabolic events may occur across diverse patient groups [6]. Contemporary reviews underscore the expanding role of SGLT2 inhibitors in cardiovascular care while also calling attention to the need for awareness of metabolic complications [7]. Mechanistic studies continue to refine understanding of how these agents influence cardiorenal and metabolic pathways, including ketone metabolism [8].

Most reported cases of euglycemic ketoacidosis have occurred in patients with diabetes, often in the setting of precipitating factors such as infection, surgery, fasting, dehydration, alcohol use, ketogenic diet, or insulin deficiency. In contrast, our patient had no history of diabetes, preserved endogenous insulin secretion, preserved kidney function, normal thyroid testing, no evidence of chronic liver disease, and no clear precipitating trigger. She also had a low-normal body mass index, a factor that may reduce metabolic reserve and has been proposed as a potential vulnerability in patients receiving SGLT2 inhibitors. This suggests that SGLT2 inhibition alone, possibly in combination with physiologic stress from tachyarrhythmia and underlying cardiac disease, may be sufficient to induce ketosis in susceptible individuals.

An additional distinguishing feature of this case is the relatively mild clinical course and rapid resolution without insulin therapy. Standard management of diabetic ketoacidosis includes insulin infusion to suppress ketogenesis; however, in this patient, preserved endogenous insulin secretion likely allowed for effective suppression of ketone production with carbohydrate supplementation alone. This observation suggests that management strategies may be individualized in carefully selected patients, particularly those without diabetes and with improving metabolic parameters.

The question of future SGLT2 inhibitor therapy requires individualized risk-benefit assessment. In patients with heart failure, these agents provide important morbidity and mortality benefits; however, in this patient, the episode occurred in the absence of modifiable precipitants. Rechallenge would therefore carry a clinically meaningful risk of recurrent ketoacidosis and could expose the patient to a similar event without a clearly correctable trigger. For this reason, we recommended permanent discontinuation of dapagliflozin and avoidance of other SGLT2 inhibitors. If future circumstances created an unusually compelling cardiorenal indication, reconsideration would require shared decision-making, close coordination with cardiology and endocrinology, education regarding sick-day rules, avoidance of fasting or ketogenic dieting, ketone monitoring during illness, and immediate drug discontinuation if ketosis or reduced intake occurred. In this case, however, the preferred strategy was optimization of alternative guideline-directed heart failure therapy rather than SGLT2 inhibitor rechallenge.

**Figure 1:**
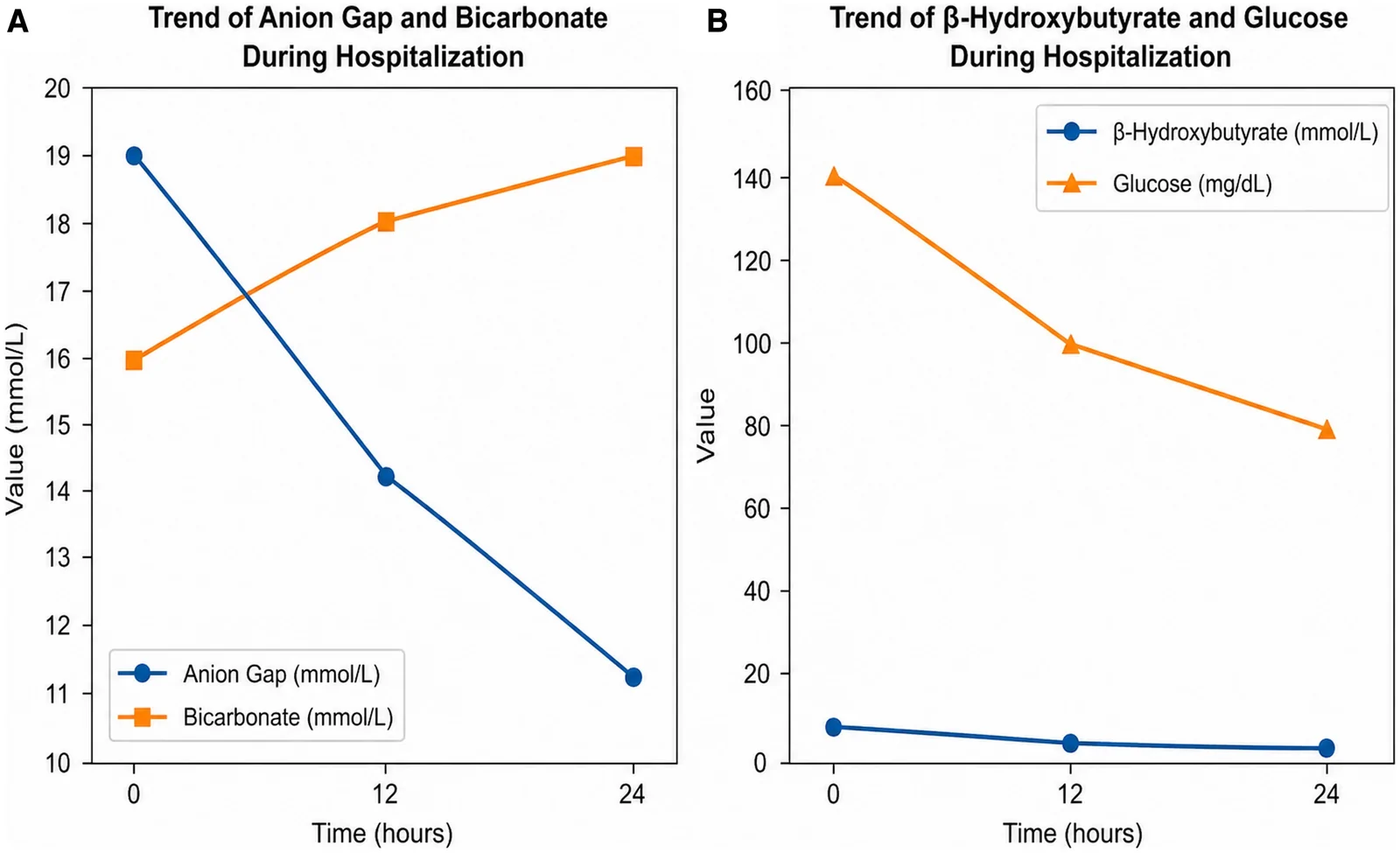
Biochemical trends during hospitalization. (A) Anion gap and bicarbonate improved. (B) Beta-hydroxybutyrate decreased with stable glucose levels.

As illustrated in Fig. 1, biochemical trends demonstrated progressive resolution of metabolic acidosis and ketosis following discontinuation of dapagliflozin and initiation of supportive therapy. The anion gap normalized, bicarbonate levels improved, and beta-hydroxybutyrate declined, while glucose levels remained within the normal range. These findings reinforce the importance of early recognition and targeted management.

This case adds to the growing body of literature describing SGLT2 inhibitor-associated euglycemic ketoacidosis in nondiabetic populations and highlights several clinically relevant points: the need for a high index of suspicion in patients with unexplained high anion gap metabolic acidosis, the importance of measuring serum ketones even in the absence of hyperglycemia, the importance of assessing for precipitating factors and metabolic vulnerabilities, and the potential for conservative management in selected cases. Further studies are needed to better define risk factors, recurrence risk after rechallenge, and optimal management strategies in this population.

## Learning points

- Euglycemic ketoacidosis is an important complication of sodium–glucose cotransporter 2 inhibitor therapy and can occur in patients without diabetes.
- The absence of marked hyperglycemia may delay recognition; clinicians should consider this diagnosis in any patient on SGLT2 inhibitors presenting with high anion gap metabolic acidosis.
- Measurement of serum ketones, particularly beta-hydroxybutyrate, is essential for diagnosis when glucose levels are normal or only mildly elevated.
- In selected patients with preserved endogenous insulin secretion and mild clinical presentation, conservative management with discontinuation of the offending agent, fluid resuscitation, and carbohydrate supplementation may be sufficient without insulin therapy.
- Increasing use of SGLT2 inhibitors for cardiovascular and renal indications requires heightened awareness of atypical metabolic adverse effects across specialties.

## Contributors

All authors made substantial contributions to the preparation of this manuscript. G.S., Y.K., and P.S. were involved in patient care, data acquisition, and manuscript drafting. H.S. and J.G. contributed to interpretation of clinical data and critical revision of the manuscript. All authors reviewed and approved the final version.

## Data Availability

All data relevant to this case report are included in this article. Additional deidentified information may be available from the corresponding author upon reasonable request and with appropriate institutional approval.
